# Bone marrow lesion volume reduction is not associated with improvement of other periarticular bone measures: data from the Osteoarthritis Initiative

**DOI:** 10.1186/ar4336

**Published:** 2013-10-16

**Authors:** Jeffrey B Driban, Grace H Lo, Lori Lyn Price, Jincheng Pang, Eric Miller, Robert J Ward, David J Hunter, Charles B Eaton, John A Lynch, Timothy E McAlindon

**Affiliations:** 1 Division of Rheumatology, Tufts Medical Center, 800 Washington Street, Box 406, Boston, MA 02111, USA; 2 Medical Care Line and Research Care Line, Houston Health Services Research and Development (HSR&D) Center of Excellence Michael E. DeBakey VAMC, Houston, TX, USA; 3 Section of Immunology, Allergy, and Rheumatology, Baylor College of Medicine, Houston, TX, USA; 4 The Institute for Clinical Research and Health Policy Studies, Tufts Medical Center, 800 Washington Street, Box 63, Boston, MA 02111, USA; 5 Tufts Clinical and Translational Science Institute, Tufts University, 800 Washington Street, Box 63, Boston, MA 02111, USA; 6 Department of Electrical and Computer Engineering, Tufts University, 101A Halligan Hall, Medford, MA 02155, USA; 7 Department of Radiology, Tufts Medical Center, 800 Washington Street, Box 299, Boston, MA 02111 USA; 8 Royal North Shore Hospital, Rheumatology Department and University of Sydney, Sydney, NSW, Australia; 9 Center for Primary Care and Prevention, Alpert Medical School of Brown University, Pawtucket, RI, USA; 10 Department of Epidemiology and Biostatistics, University of California at San Francisco, 185 Berry Street, Lobby 5, Suite 5700, San Francisco, CA 94107, USA

## Abstract

**Introduction:**

We evaluated the associations between bone marrow lesion (BML) volume change and changes in periarticular bone mineral density (paBMD) as well as subchondral sclerosis to determine whether BML change is associated with other local bone changes.

**Methods:**

The convenience sample comprised participants in the Osteoarthritis Initiative (OAI) with weight-bearing posterior-anterior knee radiographs and magnetic resonance images (MRIs) at the 24- and 48-month visits and dual-energy x-ray absorptiometry (DXA) at the 30-/36-month and 48-month visits. The right knee was assessed unless contraindicated for MRI. We used knee DXA scans to measure medial tibia paBMD and medial/lateral paBMD ratio (M:L paBMD). Knee radiographs were scored for sclerosis (grades 0 to 3) in the medial tibia. Two raters determined BML volume on sagittal fat-suppressed MRI by using a semiautomated segmentation method. To focus on knees with only medial tibia BML changes, knees with lateral tibial BMLs were excluded. Medial tibial BML volume change was classified into three groups: BML regression (lowest quartile of medial tibial BML volume change), no-to-minimal change (middle two quartiles), and BML progression (highest quartile). We used proportional odds logistic regression models to evaluate the association between quartiles of changes in medial paBMD or M:L paBMD ratio, as outcomes, and BML volume change.

**Results:**

The sample (*n* = 308) included 163 (53%) female subjects, 212 (69%) knees with radiographic osteoarthritis, and participants with a mean age of 63.8 ± 9.3 years and mean body mass index of 29.8 ± 4.7 kg/m^2^. We found an association between greater increases in medial tibia paBMD and BML regression (OR = 1.7 (95% confidence interval (CI) = 1.1 to 2.8)) and a similar trend for BML progression (OR = 1.6 (95% CI = 1.0 to 2.6]). We also detected associations between greater increase in M:L paBMD and BML regression (OR = 1.6 (95% CI = 1.0 to 2.7]) and BML progression (OR = 1.8 (95% CI = 1.1 to 3.0)), although BML regression had borderline statistical significance. The frequency of sclerosis progression in the medial tibia (*n* = 14) was greater among knees with BML progression or regression compared with knees without BML change (*P* = 0.01 and *P* = 0.04, respectively).

**Conclusion:**

BML regression and BML progression are characterized by concurrent increases in paBMD and sclerosis, which are characteristic of increased radiographic osteoarthritis severity. At least during 24 months, BML regression is not representative of improvement in other periarticular bone measures.

## Introduction

Bone marrow lesions (BMLs) are common magnetic resonance (MR) imaging findings among knees with osteoarthritis. BMLs are characterized as ill-defined regions of high-signal intensity within the subchondral bone on fluid-sensitive MR images that are associated with altered bone quality (for example, increased bone volume fraction [1-3], increased periarticular bone mineral density (paBMD) [4], decreased mineral content [2], fibrosis [3], and edema [5]). BMLs are clinically meaningful because they are associated with knee pain and disease severity (for example, cartilage damage) as well as predictive of changes in knee pain and structural progression (for example, cartilage loss) [6,7]. In recent years, it has been suggested that BML size may be an important imaging biomarker for knee osteoarthritis [8] and that reducing BML size (BML regression) may represent an important therapeutic goal for modifying osteoarthritis progression (for example, preventing or slowing joint-space narrowing) [9,10].

One limitation to adopting BML change as an imaging outcome for knee osteoarthritis is that the relation between BML changes and knee osteoarthritis progression, as well as other bone changes, remains poorly understood. Recent evidence suggests that decreases in or resolution of BMLs (BML regression) is not associated with decreased concurrent cartilage loss [6] or joint-space narrowing, and may be associated with greater odds of joint-space narrowing [6,11]. Based on these findings, we hypothesize that BML change may not reflect the full extent of pathologic changes within the periarticular bone. Therefore, it may be advantageous to determine the association between BML volume change and concurrent changes in other measures of periarticular bone. One ideal imaging marker of periarticular bone for testing this hypothesis is paBMD, which cross-sectionally is elevated with greater disease severity or intraarticular pathology (for example, joint-space narrowing [12-15], osteophytes [14,15], BMLs [4], meniscal pathology [16], sclerosis [13,15]) and decreases in the medial tibiofemoral compartment after unloading (for example, high-tibial osteotomies) [17]. Subchondral sclerosis is also a long-recognized finding in knee osteoarthritis and represents pathology in the periarticular bone. Therefore, we evaluated the associations between BML volume change in the medial proximal tibia, measured by using a validated method [18], and changes in paBMD and radiographic scoring of subchondral sclerosis. More specifically, we assessed changes in proximal medial tibia paBMD [12], which is an absolute change in paBMD, as well as change in a medial-to-lateral paBMD ratio (M:L paBMD), which is a relative change in local paBMD normalized to paBMD from a less-affected region of the proximal tibia [4,13,16]. Our primary hypothesis was that BML volume change would have a linear relation with changes in medial tibia paBMD and M:L paBMD; hence, an increase in BML volume (BML progression) would be associated with concurrent increases in medial tibia and M:L paBMD. Our secondary hypothesis was the BML regression would be associated with a reduced frequency of progression in sclerosis scores, whereas BML progression would be characterized by a greater frequency of knees with progression in sclerosis scores.

## Methods

### Participant selection

**Figure 1:**
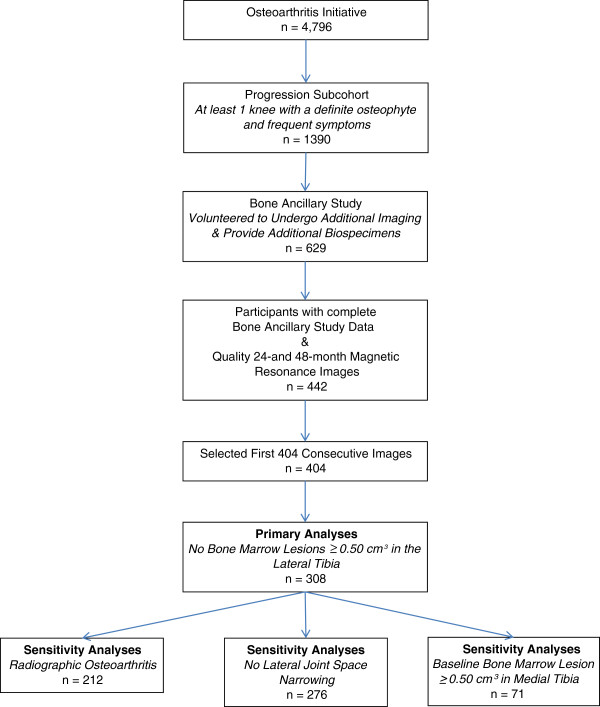
**Selection of analytic set from the Osteoarthritis Initiative.**

To assess the relation between changes in BML volume and periarticular bone we selected a convenience sample from the Osteoarthritis Initiative (OAI, *n* = 4,796); specifically the progression subcohort (*n* = 1,390; Figure 1). The progression subcohort included participants with symptomatic radiographic knee osteoarthritis in at least one knee; defined as a knee with a definite osteophyte (Osteoarthritis Research Society International (OARSI) atlas [19] osteophyte grade 1 to 3) and “pain, aching or stiffness on most days of the month in the last year”.

Within the OAI progression subcohort, the Bone Ancillary Study recruited participants (*n* = 629) during their 30- or 36-month OAI visits. The inclusion criterion for this ancillary study was a willingness to undergo additional knee MR imaging and dual-energy x-ray absorptiometry (DXA). Exclusion criteria were contraindication for MR imaging or the presence of bilateral knee replacements. For these analyses, we focused on participants in the Bone Ancillary Study with quality magnetic resonance (MR) imaging at the 24- and 48-month OAI visits (*n* = 442) and selected the first 404 knees as a convenience sample.

This study received ethical approval from each OAI clinical site (Memorial Hospital of Rhode Island Institutional Review Board, The Ohio State University’s Biomedical Sciences Institutional Review Board, University of Pittsburgh Institutional Review Board, and University of Maryland Baltimore–Institutional Review Board), the OAI coordinating center (Committee on Human Research at University of California, San Francisco), and the Institutional Review Board at Tufts Medical Center and Tufts University Health Sciences Campus. All participants provided informed consent to the OAI and the Bone Ancillary Study.

### Magnetic resonance imaging

We evaluated BML volume on sagittal intermediate-weighted, turbo spin-echo, fat-suppressed MR sequences (field of view = 160 mm, slice thickness = 3 mm, skip = 0 mm, flip angle = 180 degrees, echo time = 30 ms, recovery time = 3,200 ms, 313 × 448 matrix (interpolated to 512 × 512), phase encode superior/inferior, x resolution = 0.357 mm, and y resolution = 0.511 mm). Images were acquired at each of the four OAI clinical sites with a Siemens Trio 3-Tesla MR system with a USA Instruments quadrature transmit-receive knee coil at the 24- and 48-month OAI visits.

We focused on the primary OAI knee, which was the right knee unless there was a contraindication, in which case, the left knee was the primary knee. Therefore, the primary knee was not always the knee with symptomatic osteoarthritis. According to the OAI protocol, the primary knee underwent a complete set of OAI MR sequences, whereas the contralateral knee underwent an abbreviated MR scan to reduce participant burden.

### Semiautomated BML segmentation

We used a semiautomated segmentation method to determine BML volume change. We previously demonstrated the validity of this method with OAI images by demonstrating that increases in BML volume were associated with cartilage loss, and BML volumes differed across Boston Leeds Osteoarthritis Knee Score [18]. A detailed description of the segmentation method may be found in [18].

In brief, two readers measured BML volume with a semiautomated segmentation method. The only manual step required a reader to use a graphic user interface (MATLAB; MathWorks, Inc., Natick, MA, USA) to identify the crude boundaries of the tibia and femur in each slice of the MR sequence by marking points along the articular surface. For the border farthest from the articular surface, the reader marked the bone just before the epiphyseal line or at the edge of bone and soft tissue. We omitted the central slices from the analyses (that is, the middle nine slices; 2.7 cm), which corresponded to the subspinous region in the tibia, to focus on BMLs adjacent to the tibiofemoral chondral surface and to improve reliability. The central region was automatically detected after the reader manually marked the most medial and lateral MR images that included the femoral condyles. The program automatically refined the initial bone border to identify more precisely the bone boundaries. Next, the program automatically applied a thresholding and curve evolution process twice to segment the areas of high-signal intensity, which represent a probable BML. We then used two criteria to eliminate false positive regions and further to define a BML: (1) the distance between a BML and the articular surface should be ≤10 mm [20]; and (2) a BML should appear on adjacent images. BML volumes were calculated for the medial and lateral tibia.

With images from the OAI (*n* = 10 or 12 knees), we found good intrareader (intraclass correlation coefficient (ICC) model 3,1 = 0.79 to 0.99) and interreader reliability (ICC 2,1 model = 0.59 to 0.93) for BML volume change [18]. To ensure consistency between readers, a third investigator reviewed all of the BML segmentations to verify that the bone segmentation was consistent across time and knees.

### Dual-energy X-ray absorptiometry

To evaluate paBMD, the proximal tibia was scanned by using DXA (Lunar Prodigy Advance scanner; GE Lunar Corp., Madison, WI, USA), with a customized knee-analysis software option. DXA scans were acquired at the 30- or 36-month and 48-month OAI visits. A standard protocol [12] was used to ensure that the lower extremity was positioned and stabilized consistently across OAI clinical sites. A positioning laser light was used to center the scanner arm 5 cm below the inferior pole of the patella.

### Periarticular bone mineral density

One analyst performed the paBMD measurements. The regions of interest (ROIs) were 10 mm vertically, and the mediolateral (horizontal) direction was half the distance between the medial and lateral bone edges (creating two ROIs: medial paBMD and lateral paBMD). The ROIs were positioned so that the top borders were just superior and parallel to the joint surfaces of the tibia. For each ROI, the paBMD was measured in the area bounded by the bone edges and the boundaries of the ROI positioned within the bone. A paBMD ratio was derived by dividing the medial paBMD by the lateral paBMD (M:L paBMD). The test-retest (with repositioning) intraclass correlation was 0.99 for the tibial paBMD (*n* = 10). More-specific details regarding the reliability of these measurements in this data set have been previously described [12].

### Knee radiographs

Bilateral, weight-bearing, fixed-flexion, posterior-anterior knee radiographs were obtained at the 24- and 48-month OAI visits. Readers, who were blinded to sequence, scored the paired images for Kellgren-Lawrence grade (0 to 4) as well as medial tibial sclerosis grade (0 to 3) and joint space narrowing by using the OARSI Atlas [19]. The agreement for these readings (read-reread) was good (weighted kappa, ≥0.75). Radiographic scores are publicly [21] (Files: kXR_SQ_BU03_SAS [version 3.4] and kXR_SQ_BU06_SAS [version 6.2]).

### Preliminary analyses

To determine a BML volume threshold for defining a region with a relevant BML volume, we conducted a proportional odds logistic regression to determine the cross-sectional association (48-month visit) between BML volume and medial tibial paBMD. The outcome was medial tibial paBMD, stratified in quartiles, and the predictor was medial tibial BML volume, stratified in quartiles. The model was adjusted for age (<65 years, ≥65 years) and obesity (body mass index, <30 kg/m^2^, ≥30 kg/m^2^). Odds ratios (ORs) and 95% confidence intervals (CIs) were computed to compare the upper three quartiles with the lowest quartile of BML volume (BML volume <0.06 cm^3^).

The second and third quartiles of BML volume did not have greater medial tibial paBMD than the first quartile (second quartile OR = 1.59; 95% CI = 0.89 to 2.84; third quartile OR = 1.50; 95% CI = 0.84 to 2.67). However, the fourth quartile of BML volume (BML volumes >0.46 cm^3^) was associated with a higher medial tibial paBMD than the first quartile (OR = 3.35; 95% CI = 2.07 to 4.86). Therefore, we adopted an estimate that a BML volume > 0.50 cm^3^ represented a region with a relevant BML volume. Thus, we excluded knees with lateral tibial BML volumes >0.50 cm^3^ (at 24- or 48-month OAI visits) because we wanted to ensure that the lateral tibia was a good reference region for the M:L paBMD ratio, particularly because we were interested in changes in the medial tibia.

### Statistical analyses

We calculated descriptive statistics to characterize this study population by using data that are publicly [21] (Files: allclinical (version 0.2.2, 3.2.1, 6.2.1), enrollees (version 17)). Before conducting the primary analyses, we evaluated the point estimates across quartiles of each continuous independent variable (that is, age, body mass index, and BML volume change) to verify whether they had a linear relation with the outcomes. Because age and body mass index did not have a linear relation with the outcomes, we converted them to binary variables based on common cut points. Furthermore, medial tibia BML volume change did not have a linear relation with the outcomes and was therefore classified into quartiles. We chose the middle two quartiles of BML volume change as the reference group. Therefore, medial tibial BML volume change was classified into three groups: (1) BML regression (lowest quartile of medial tibial BML volume change), (2) no-to-minimal change (middle two quartiles), and (3) BML progression (highest quartile).

Changes in medial paBMD and M:L paBMD ratio were calculated as rate of change to control for different observation periods across the cohort (for example, (follow-up data – baseline data)/(duration of observation)). We then calculated quartiles of changes in medial paBMD and M:L paBMD ratio. We used two proportional-odds logistic regression models to evaluate the association between quartiles of changes in medial paBMD or M:L paBMD ratio, as outcomes (y-variables), and change in medial tibia BML volume (classified into three groups). The models were adjusted for sex, age (<65 years, ≥65 years), and obesity (body mass index < 30 kg/m^2^, ≥30 kg/m^2^). The proportional odds assumption was met (medial paBMD: *P* = 0.97, M:L paBMD ratio: *P* = 0.17).

We replicated the primary analyses in two sets of sensitivity analyses. We limited the first sensitivity analysis to knees with radiographic knee osteoarthritis (Kellgren-Lawrence score ≥2, *n* = 212) and the second sensitivity analysis to knees without lateral joint space narrowing (*n* = 276). The proportional-odds assumption was met in these sensitivity analyses (*P* > 0.22).

A final sensitivity analysis was conducted among knees with a baseline medial tibial BML volume ≥0.50 cm^3^. We used proportional-odds logistic regression models to evaluate the association between quartiles of changes in medial paBMD or M:L paBMD, as the outcomes, and change in medial tibial BML volume (classified into three groups). Proportional odds assumption was met *P* = 0.86. These final sensitivity analyses were unadjusted because of the small sample size.

Because only a small number of knees had increased medial tibia sclerosis scores, we used Fisher Exact Tests to explore whether the frequency of knees with sclerosis progression was different between BML volume-change groups.

## Results

The baseline and longitudinal characteristics (*n* = 308, excluding those with lateral tibia BMLs) are described in Tables 1 and 2.

**Table 1:** Descriptive baseline characteristics of knees with medial tibia bone marrow lesion (BML) regression, progression, or no change

**Variable**	**BML regression (*n*** **= 76)**	**No BML or no BML change (*n*** **= 156)**	**BML progression (*n*** **= 76)**
	**Median (Min, Max) or *n *** **(%)**	**Median (Min, Max) or *n *** **(%)**	**Median (Min, Max) or *n *** **(%)**
Age (years)	65 (50, 81)	61 (48, 82)	68 (48, 82)
Body mass index (kg/m^2^)	29.3 (21.1, 40.9)	29.4 (20.1, 42.0)	29.5 (19.6, 40.7)
Female	40 (52.0%)	85 (54.5%)	38 (50.0%)
Kellgren-Lawrence grade ≥ 2	61 (80.3%)	95 (61.3%)	56 (73.7%)
Baseline BML volume (cm^3^)	0.55 (0.16, 10.98)	0.10 (0.01, 2.94)	0.15 (0.01, 3.51)
Baseline medial tibia paBMD	1.129 (0.681, 1.890)	1.145 (0.761, 1.743)	1.111 (0.800, 1.664)
Baseline M:L paBMD ratio	1.138 (0.799, 1.549)	1.096 (0.794, 1.779)	1.155 (0.914, 1.385)
Number of knees with baseline BML volume ≥0.50 cm^3^	43 (56%)	7 (4%)	21 (27%)

Notes: paBMD, periarticular bone mineral density; M:L paBMD ratio, medial-to-lateral paBMD ratio.

**Table 2:** Descriptive longitudinal characteristics of knees with medial tibia bone marrow lesion (BML) regression, progression, or no change

**Variable**	**BML regression (*n*** **= 76)**	**No BML or No BML change (*n*** **= 156)**	**BML progression (*n*** **= 76)**
	**Median (Min, Max) or *n *** **(%)**	**Median (Min, Max) or *n *** **(%)**	**Median (Min, Max) or *n *** **(%)**
BML volume change (cm^3^)	-0.36 (-8.44, -0.14)	-0.02 (-0.13, 0.03)	0.21 (0.04, 6.77)
Medial tibia paBMD change (g/cm^2^)	0.002 (-0.149, 0.164)	-0.011 (-0.107, 0.149)	-0.001 (-0.091, 0.168)
M:L paBMD ratio (change)	0.005 (-0.119, 0.291)	-0.007 (-0.102, 0.091)	0.003 (-0.080, 0.410)
Medial tibia sclerosis progression	5 (7.0%)	2 (1.3%)	7 (10.0%)

Notes: paBMD, periarticular bone mineral density; M:L paBMD ratio, medial-to-lateral paBMD ratio.

### BML volume changes are associated with paBMD changes

We found an association between greater BML regression and medial tibia paBMD increases (odds ratio (OR) = 1.72) and a similar trend for BML progression (OR = 1.61; Table 3). We also detected a similar association of BML regression (OR = 1.64) and BML progression (OR = 1.85) with increases in M:L paBMD ratio, although BML regression had borderline statistical significance (Table 4).

**Table 3:** **Distribution of medial periarticular bone mineral density (paBMD) changes among knees with medial tibia bone marrow lesion (BML) regression, progression, or no change (*n*** **= 308)**

	**Outcome: Medial paBMD change quartiles (min, max, gm/cm**^**2**^**)**				
**Predictor**	**1st Quartile**	**2nd Quartile**	**3rd Quartile**	**4th Quartile**	**Proportional odds ratio**
	**(-0.149, -0.033)**	**(-0.032, -0.004)**	**(-0.004, 0.018)**	**(0.018, 0.168)**	
	***n*** **= 77**	***n*** = **77**	***n*** **= 77**	***n*** **= 77**	**(95% CI)**
BML Regression (-8.44, -0.14 cm^3^) *n* = 76	16 (21%)	15 (20%)	22 (29%)	23 (30%)	1.72 (1.05 - 2.82)
No BML or no BML change (-0.13, 0.03 cm^3^) *n* = 156	45 (29%)	44 (28%)	36 (23%)	31 (20%)	Reference
BML Progression (0.04, 6.77 cm^3^) *n* = 76	16 (21%)	18 (24%)	19 (25%)	23 (30%)	1.61 (0.98 - 2.65)

Notes: paBMD, periarticular bone mineral density. Proportional odds ratio of greater quartile of medial paBMD is compared with the referent group.

**Table 4:** **Distribution of medial-to-lateral periarticular bone mineral density (M:L paBMD) changes among knees with medial tibia bone marrow lesion (BML) regression, progression, or no change (*n*** **= 308)**

	**Outcome: Medial:lateral paBMD ratio change quartiles (min, max)**				
**Predictor**	**1st Quartile**	**2nd Quartile**	**3rd Quartile**	**4th Quartile**	**Proportional odds ratio**
	**(-0.119, -0.021)**	**(-0.021, 0.000)**	**(0.000, 0.022)**	**(0.022, 0.410)**	
	***n*** **= 77**	***n*** **= 77**	***n*** **= 77**	***n*** **= 77**	**(95% CI)**
BML Regression (-8.44, -0.14 cm^3^) *n* = 76	14 (18%)	18 (24%)	25 (33%)	19 (25%)	1.64 (1.00 – 2.69)
No BML or no BML change (-0.13, 0.03 cm^3^) *n* = 156	47 (30%)	45 (29%)	30 (19%)	34 (22%)	Reference
BML Progression (0.04, 6.77 cm^3^) *n* = 76	16 (21%)	14 (18%)	22 (29%)	24 (32%)	1.85 (1.12 – 3.04)

Notes: paBMD, periarticular bone mineral density. Proportional odds ratio of greater quartile of medial paBMD is compared with the referent group.

Sensitivity analyses, conducted among knees with radiographic knee osteoarthritis (*n* = 212), supported the primary results. We found an association between greater medial tibia paBMD change and BML regression (OR = 2.07; 95% CI = 1.15 to 3.73) and BML progression (OR = 1.83; 95% CI, 1.00 to 3.36). Similarly, greater M:L paBMD ratio change showed a similar trend with BML regression (OR = 1.74; 95% CI, 0.97 to 3.12) and BML progression (OR = 1.90; 95% CI = 1.03 to 3.49).

Sensitivity analyses, conducted among knees without lateral joint-space narrowing (*n* = 276), also supported the primary results. We found an association between greater medial tibia paBMD change and BML regression (OR = 1.78; 95% CI, 1.05 to 3.03) and BML progression (OR = 1.68; 95% CI, 1.00 to 2.82]). Similarly, greater M:L paBMD ratio change had a similar trend with BML regression (OR = 1.55; 95% CI, 0.91 to 2.63) and BML progression (OR = 1.79; 95% CI, 1.06 to 3.01]).

Sensitivity analyses, conducted among knees with a baseline medial tibial BML volume ≥0.50 cm^3^ (*n* = 71), supported the primary results for medial tibia paBMD change but not M:L paBMD change (*P* = 0.87). We found an association between greater medial tibia paBMD change and BML regression (OR = 5.97; 95% CI, 1.28 to 27.86) and BML progression (OR = 7.44; 95% CI, 1.43 to 38.68).

### BML volume changes are associated with sclerosis progression

Exploratory analyses indicated that the frequency of sclerosis progression in the medial tibia was greater among knees with BML progression or regression compared with knees with no BML change (Table 2; *P* = 0.01 and *P* = 0.04, respectively).

## Discussion

Bone marrow lesions are associated with regions of increased paBMD [4], which is related to greater osteoarthritis severity (for example, joint-space narrowing, subchondral sclerosis) [13]. We found that compared with knees with no BML change, BML progression and BML regression were associated with increased paBMD and sclerosis in the medial tibia, reflective of radiographic osteoarthritis progression. These findings may provide an explanation for why a decrease in BML volume is not associated with decreased odds of structural progression (for example, cartilage loss, joint-space narrowing) [6,11]. Overall, these analyses suggest that BML regression may not represent a concurrent improvement in other periarticular bone measures (that is, subchondral sclerosis and paBMD).

These findings improve our understanding of BMLs and BML changes. Bone marrow lesions are characterized as areas of increased bone-volume fraction [1-3], increased paBMD [4], decreased mineral content [2], fibrosis [3], edema [5], and necrosis, which can change size in short periods (for example, 6 to 12 weeks) [8]. The rapid changes in BML size, which have been previously reported [8], may correspond to changes in bone marrow (for example, edema, fibrosis) that could be associated with changes in knee pain [7,22], but not changes that influence bone morphometry (for example, trabecular morphometry, bone mineral density, bone mineral content), which may take longer to remodel. Therefore, the MR-imaging signal associated with changes in BML may be an imaging biomarker that is associated with immediate changes in pain stimuli but not overall bone. It is important to note that conflicting data exist on the relation between BML size and knee pain; however, recent systematic reviews support this association [7,22]. Reducing BML size may be an important therapeutic target for decreasing knee pain, but thus far, its importance in modifying structural progression remains doubtful over the short term (<2 years) and requires additional research for longer observation periods.

This study offers new insights into BMLs, but it is important to note that we used an apparent measure of bone density that included cortical bone. It may be advantageous for future studies to explore this question with measures of trabecular morphometry along with MR spectroscopic imaging, which may detect changes in the bone marrow. Future studies will also be needed to understand whether BML regression eventually leads to improved bone measures and decreased risk of osteoarthritis progression over the long term (for example, cartilage loss, joint-space narrowing). These studies may also require more-frequent imaging to determine when the BML changes are occurring because it is unclear in these analyses if the BML change occurred gradually over a 2–year period or in short periods during the observation period. More-frequent assessments may also address the implications of bone exposed to repeated appearances of BMLs. The utility of BML change as an outcome in clinical trials and as a potential target for disease-modifying interventions will remain doubtful until these lingering issues are addressed.

Another limitation to our study was that we had a small sample size of individuals with baseline BML volume ≥0.50 cm^3^ (*n* = 71) and changes in sclerosis scores (*n* = 14). Despite this small sample size, we were able to detect longitudinal associations in unadjusted analyses that supported the primary results. Future studies could try to pursue a larger sample size to address these analyses or opt for direct assessments of bone.

## Conclusions

We propose that BML regression and BML progression are characterized by concurrent increases in paBMD and sclerosis, which are characteristic of increased radiographic osteoarthritis severity. At least during 24 months, it appears that BML regression is not representative of improvement in other bone measures. Therefore, BML change may represent a transient phase of the natural history of periarticular bone changes in OA.

## Abbreviations

BML: Bone marrow lesion; CI: Confidence interval; DXA: Dual-energy x-ray absorptiometry; ICC: Intraclass correlation coefficient; M:L paBMD: Medial-to-lateral periarticular bone-mineral density; MR: Magnetic resonance; OAI: Osteoarthritis Initiative; OARSI: Osteoarthritis research society international; OR: Odds ratio; paBMD: Periarticular bone-mineral density; ROI: Region of interest.

## Competing interests

The authors declare that they have no competing interests.

## Authors’ contributions

JBD contributed to the conception and design, acquisition of data, analysis and interpretation of data, drafting/revisions of the article, as well as final approval of the article. GHL contributed to the conception and design, analysis and interpretation of data, drafting/revisions of the article, as well as final approval of the article. LLP contributed to the conception and design, analysis and interpretation of data, drafting/revisions of article, as well as final approval of the article. JP contributed to the conception and design, acquisition of data, analysis and interpretation of data, drafting/revisions of article, as well as final approval of the article. EM contributed to the conception and design, acquisition of data, analysis and interpretation of data, drafting/revisions of the article, as well as final approval of the article. RW contributed to the conception and design, acquisition of data, analysis and interpretation of data, drafting/revisions of the article, as well as final approval of the article. DJH contributed to the conception and design, acquisition of data, analysis and interpretation of data, drafting/revisions of the article, as well as final approval of the article. CBE contributed to the conception and design, analysis and interpretation of data, drafting/revisions of the article, as well as final approval of the article. JAL contributed to the conception and design, acquisition of data, analysis and interpretation of data, drafting/revisions of the article, as well as final approval of the article. TEM contributed to the conception and design, analysis and interpretation of data, drafting/revisions of the article, as well as final approval of the article. All authors read and approved the final manuscript.
